# Novel Mutations Within Collagen Alpha1(I) and Alpha2(I) Ligand-Binding Sites, Broadening the Spectrum of Osteogenesis Imperfecta – Current Insights Into Collagen Type I Lethal Regions

**DOI:** 10.3389/fgene.2021.692978

**Published:** 2021-07-09

**Authors:** Kinga Sałacińska, Iwona Pinkier, Lena Rutkowska, Danuta Chlebna-Sokół, Elżbieta Jakubowska-Pietkiewicz, Izabela Michałus, Łukasz Kępczyński, Dominik Salachna, Aleksander Jamsheer, Ewelina Bukowska-Olech, Ilona Jaszczuk, Lucjusz Jakubowski, Agnieszka Gach

**Affiliations:** ^1^Department of Genetics, Polish Mother’s Memorial Hospital Research Institute, Łódź, Poland; ^2^Department of Bone Metabolic Diseases, University Centre of Paediatric, Medical University of Łódź, Łódź, Poland; ^3^Department of Paediatric Propedeutics and Bone Metabolic Diseases, Medical University of Łódź, Łódź, Poland; ^4^Department of Medical Genetics, Poznan University of Medical Sciences, Poznań, Poland; ^5^Centers for Medical Genetics GENESIS, Poznań, Poland; ^6^Department of Cancer Genetics with Cytogenetics, Medical University of Lublin, Lublin, Poland

**Keywords:** osteogenesis imperfecta, *COL1A1*, *COL1A2*, genotype–phenotype correlation, next generation sequencing, fractures, collagen type I, genetic background

## Abstract

Osteogenesis imperfecta (OI) is a rare genetic disorder demonstrating considerable phenotypic and genetic heterogeneity. The extensively studied genotype–phenotype correlation is a crucial issue for a reliable counseling, as the disease is recognized at increasingly earlier stages of life, including prenatal period. Based on population studies, clusters in *COL1A1* and *COL1A2* genes associated with the presence of glycine substitutions leading to fatal outcome have been distinguished and named as “lethal regions.” Their localization corresponds to the ligand-binding sites responsible for extracellular interactions of collagen molecules, which could explain high mortality associated with mutations mapping to these regions. Although a number of non-lethal cases have been identified from the variants located in lethal clusters, the mortality rate of mutations has not been updated. An next generation sequencing analysis, using a custom gene panel of known and candidate OI genes, was performed on a group of 166 OI patients and revealed seven individuals with a causative mutations located in the lethal regions. Patients’ age, ranging between 3 and 25 years, excluded the expected fatal outcome. The identification of non-lethal cases caused by mutations located in lethal domains prompted us to determine the actual mortality caused by glycine substitutions mapping to lethal clusters and evaluate the distribution of all lethal glycine mutations across collagen type I genes, based on records deposited in the OI Variant Database. Finally, we identified six glycine substitutions located in lethal regions of *COL1A1* and *COL1A2* genes, of which four are novel. The review of all mutations in the dedicated OI database, revealed 33 distinct glycine substitutions in two lethal domains of *COL1A1*, 26 of which have been associated with a fatal outcome. Similarly, 109 glycine substitutions have been identified in eight lethal clusters of *COL1A2*, of which 51 have been associated with a fatal manifestation. An analysis of all glycine substitutions leading to fatal phenotype, showed that their distribution along collagen type I genes is not regular, with 17% (26 out of 154) of mutations reported in *COL1A1* and 64% (51 out of 80) in *COL1A2* corresponding to localization of the lethal regions.

## Introduction

Osteogenesis imperfecta (OI) is a rare connective tissue disorder with considerable clinical and genetic heterogeneity. The extensively studied genotype–phenotype correlation is a crucial issue for a reliable counseling, as with the advances in ultrasound scans the disease is increasingly recognized at prenatal period. The mechanism of pathogenesis is predominantly linked with the *COL1A1* and *COL1A2* genes, whose mutations result in quantitative or qualitative defects of collagen alpha(I) chains (Kang et al., 2017; Robinson and Rauch, 2019; Steiner and Basel, 2019). Alterations of collagen type I molecules affect a number of tissues, such as bone, ligament, and tendon, as well as cartilage, dermis, dentine, sclera, and cornea, resulting in various skeletal and extra-skeletal manifestations. The most characteristic features of OI are multiple low-energy fractures, short stature, long bones and chest deformations, triangular-shaped face, blue sclera, abnormalities of the dentin (Dentinogenesis imperfecta, DI), hearing impairment, and joint hypermobility.

Depending on clinical spectrum and severity, four basic OI types ranging from mild to perinatal lethal have been originally distinguished. They are all associated with mutations in the *COL1A1* and *COL1A2* genes. Type I is the mildest form of the disease. Patients are characterized by normal or nearly normal height, blue sclera, joint hypermobility, and hearing impairment, which occurs in adulthood. Increased susceptibility to fractures, not resulting in bone deformations, often occurs at birth or when the child begins to walk, but decreases after puberty. Type II is the most severe form, characterized by multiple *in utero* fractures, shortened and bowed limbs, flexed and abducted hips, a small chest with beaded ribs, hypoplastic face, large and soft calvarium, deep blue sclera, and osteopenia. Due to numerous skeletal deformations, the majority of children with type II are stillborn or die in the perinatal period because of respiratory problems. Type III, or progressively deforming, is the most severe non-lethal form of OI apparent from birth. The majority of patients have to use a wheelchair due to severe bone fragility, a barrel-shaped chest, and deformations of the long bones. Facial dysmorphism, marked short stature, and basilar impression are commonly observed. Type IV is a moderate form, with clinical features demonstrating severity between mild type I and progressively deforming type III (Cole and Dalgleish, 1995; Basel and Steiner, 2009). Current nosology distinguishes additional fifth OI type presenting hyperostosis, mapping to *IFITM5* gene. The suggested subdivision corresponds to the severity of symptoms, based on clinical and radiological features, without taking into account the genetic background (Mortier et al., 2019). On the contrary, the Online Mendelian Inheritance in Men database distinguishes XXI types of OI, where types VI–XXI result from pathogenic variants located in distinct non-collagen genes, however, presenting clinical features overlapping with type II, III, and IV (Van Dijk and Sillence, 2014; Trejo and Rauch, 2016; Online Mendelian Inheritance in Men (OMIM), 2021).

Nowadays, it is possible to identify fractures and bone deformations suggesting metabolic bone disorder (e.g., OI, hypophosphatasia, thanatophoric dysplasia, campomelic dysplasia, and achondrogenesis), using ultrasound examination in the prenatal period. In case of OI, the lethal type II can be detected at 13–14 weeks of gestation, progressively deforming type III at 17–18 weeks, and mild type I later in pregnancy, when fractures or bowed femurs occur. Molecular testing can also be performed to identify the genetic cause in the fetus; this can be used to verify the primary diagnosis and even distinguish classic non-deforming OI from other types. Thus, ultrasound examination supported by DNA analysis may impact perinatal care and even influence the decision to continue the pregnancy, in the case of severe OI type II (Steiner and Basel, 2019; Online Mendelian Inheritance in Men (OMIM), 2021).

In recent years molecular testing has allowed growing numbers of individuals to be identified with causative mutations, thus enabling the determination of particular genetic factors and its potential severity. It is believed that the final clinical outcome is influenced by the gene in which the mutation is located, the type of mutation, its position in the alpha(I) chain and the particular amino acid substituting for glycine (Byers et al., 1991; Cole and Dalgleish, 1995; Bodian et al., 2008; Basel and Steiner, 2009; Marini et al., 2012; Kang et al., 2017).

Early genotype–phenotype analysis of perinatal lethal OI highlighted the absence of lethal mutation hot-spots in either the *COL1A1* or *COL1A2* gene, as the majority of mutations reported in unrelated babies were “private” and occurred *de novo*. Type II was reported to result from structural mutations, of which two-thirds were found in the alpha1(I) chain, with the preferred location toward the carboxyl-terminal end (Cole and Dalgleish, 1995). The first reports about regions containing high concentrations of lethal mutations was mentioned by Marini et al. (1993) and Wang et al. (1993). Their observations revealed an alternating occurrence of clusters containing lethal and non-lethal glycine substitutions along the gene, with three such regions present in *COL1A1* and two in *COL1A2*. This discovery indicated that the clinical severity of OI is not only determined by the impact of the mutation on the structure or assembly of the monomer, but may also play a role in higher order interactions with multiple matrix molecules (Di Lullo et al., 2002; Sweeney et al., 2008).

The mapping of the collagen type I protein revealed three Major Ligand Binding Regions (MLBR) and various functional domains in the collagen type I genes (Di Lullo et al., 2002). Although three such regions were distinguished, a strong relationship was observed between the localization of lethal mutations with MLBR1 and MLBR2 of the alpha1(I) chain (Sweeney et al., 2008; Marini et al., 2012).

In 2007, based on an analysis of over 800 mutations leading to OI type II, III, and IV, Marini et al. distinguished two stretches of glycine substitutions resulting in almost exclusively lethal outcomes at helical positions 691–823 and 910–964 in the alpha1(I) chain, referring to the localization of second and third major ligand-binding region, respectively. In the collagen alpha2(I) chain, eight regularly spaced clusters containing lethal mutations were described. They are concentrated in two-thirds of the chain and correspond to regions that play crucial roles in the interactions between collagen fibrils and proteoglycans (Marini et al., 2012). The impact of MLBRs on the final clinical outcome of mutations was also highlighted by Bodian et al. (2008).

Since the lethal regions were distinguished, the database of OI mutations has significantly expanded. A number of cases have been revealed that question whether mutations mapping to ligand-binding sites are always fatal (Pollitt et al., 2006; Bodian et al., 2009; Rauch et al., 2010; Reuter et al., 2013; Lindahl et al., 2015; Maasalu et al., 2015; Hruskova et al., 2016; Zhytnik et al., 2017, 2019; Maioli et al., 2019). Unfortunately, most of our knowledge about these special gene regions is still based on the primary findings obtained in 2007, as well as some review articles, individual publications, and separate records from the dedicated Osteogenesis Imperfecta Variant Database (OIVD; Bodian et al., 2009; Rauch et al., 2010; Mouna Ben Amor et al., 2011; Forlino et al., 2012; Marini and Blissett, 2013; Reuter et al., 2013; Maasalu et al., 2015; Forlino and Marini, 2016; Kang et al., 2017; Marini et al., 2017; Maioli et al., 2019; Osteogenesis Imperfecta Variant Database, 2020). Although multiple sources refer to lethal regions, no overall summary of related mortality is currently available. More importantly, due to the continuous expansion of the OI mutation database, the lethal regions require constant review.

**FIGURE 1:**
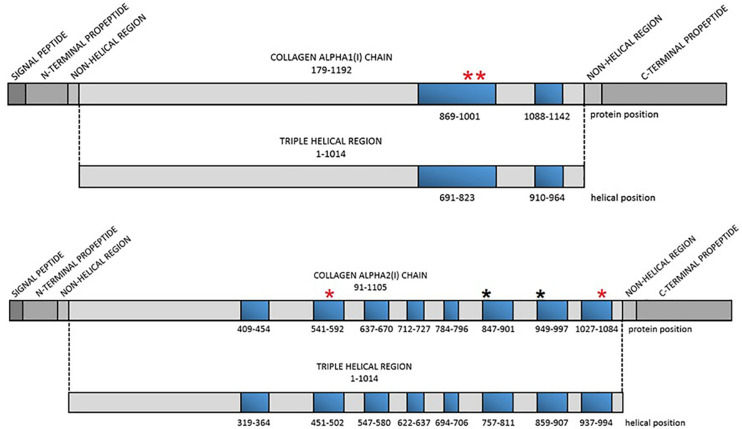
Localization of the lethal regions of collagen type I genes and the identified variants. Red asterisk refers to localization of the novel variants, black asterisk refers to localization of variants reported previously. Blue bars represent particular lethal regions. The numbering of the regions corresponds to protein and helical position in each alpha(I) chain.

The present paper is a fragment of a population-based study analyzing genetic background of OI in a Polish population using custom gene panel for next generation sequencing (NGS). Among 166 recruited individuals, we have identified a group of seven patients with causative mutations situated in the lethal regions of collagen type I genes. Despite variants location, patients presented mild, moderate, and progressively deforming OI phenotypes. Probands were found to carry one out of six glycine substitutions, of which four are novel and two has been described previously (Figure 1). The identification of mutations causing a non-lethal phenotypes placed in this specific regions prompted us to investigate the OIVD to determine what proportion of glycine substitutions within lethal domains of *COL1A1* and *COL1A2* are actually associated with a fatal outcome. The results were expanded by a short analysis of all glycine substitutions leading to lethal phenotype and their distribution along collagen type I genes regarding lethal regions.

## Materials and Methods

### Subjects

In the present paper, we describe in detail seven patients with a causative variants located in the lethal regions of collagen type I genes. Individuals were of Caucasian origin, and aged 3–25 years. Probands were identified as part of a population-based study, which included 166 patients presenting with a broad clinical manifestation of OI. The group was recruited by six Polish medical health centers. The study was approved by the local Ethics Committee. Informed consent was obtained from patients and legal guardians of children below 14 years old before the study. Clinical characteristics were derived from medical records by retrospective chart review.

### Next Generation Sequencing

Molecular testing using NGS technique was performed in the entire study group. Genomic DNA was automatically extracted from peripheral blood leukocytes using a MagCore Genomic DNA Whole Blood Kit (RBC Bioscience, Taiwan). The pathogenic variants were detected using a custom gene panel containing OI and OI-related genes (*COL1A1*, *COL1A2*, *IFITM5*, *SERPINF1*, *CRTAP*, *P3H1*, *PPIB*, *SERPINH1*, *FKBP10*, *SP7*, *BMP1*, *TMEM38B*, *WNT1*, *CREB3L1*, *SPARC*, *PLS3*, *SEC24D*, *P4HB*, *PLOD2*, *TNFSF11*, *LRP5*, *DKK1*, *RANKL*, *XYLT2*, *BMP2*, *BMP3*, *BMP4*, *BMP5*, *BMP6*, *BMP7*, *OPG*, *SOST*, *IL6*, and *IGF1*) for resequencing the exons and flanking non-coding sequences (Illumina Design Studio software). The gene panel, composed of 710 amplicons with a mean length of 175 bp, reached 99% coverage. TruSeq Custom Amplicon libraries were prepared according to the manufacturer’s protocol and sequenced on the MiniSeq platform (Illumina, United States) using two types of cartridge (Mid Output and High Output Kit, 300 cycles; Illumina, United States).

### Variant Identification, Classification, and Validation

The six described variants were identified through a multi-step bioinformatic process. The primary analysis was performed using Illumina VariantStudio software according to the GenBank human reference cDNA sequence for the *COL1A1* (NM_000088.3) and *COL1A2* (NM_000089.3) genes (GRCh37). Variant filtering and prioritization was performed using the Varsome variant interpretation tool^1^ according to the population frequency value for the European non-Finnish population (<2%) and several prediction programs. Additionally, variants were checked in public databases (dbSNP, gnomAD Exomes, gnomAD Genomes, ClinVar, HGMD, OIVD – part of LOVD) and available literature. Multiple Sequence Alignment analysis, using Clustal Omega 1.2.4 and Jalview 2.11.0 software, was performed to generate alignments between multiple sequences and analyze the conservation score of novel variants across species. Finally, the mutations were classified according to ACMG recommendations.

Any pathogenic, likely pathogenic or variants of unknown significance were selected and validated by Sanger sequencing. Custom primers were designed for regions containing the selected mutations. The PCR products were cleaned (ExoSAP-IT; Applied Biosystems, United States), labeled (BigDye Terminator v3.1 Cycle Sequencing Kit; Applied Biosystems, United States), and sequenced using a 3500 Genetic Analyzer (Thermo Fisher Scientific, United States). The obtained data was analyzed using Mutation Surveyor V5.1.0 software (SoftGenetics, United States).

### Review of all Reported Glycine Substitutions in Lethal Regions of *COL1A1* and *COL1A2* Genes

Based on the discovery of variants located within lethal regions resulting in non-lethal phenotypes, we examined over three thousand records collected in 2020 in the open online OIVD^2^ to prepare two data-analysis.

The first, concerned the clinical severity of all glycine substitutions mapping to two lethal regions of the *COL1A1* gene and eight regions of the *COL1A2* gene (according to the localization described by Marini et al.), to evaluate what proportion of glycine substitutions within lethal domains is actually associated with a fatal outcome. The second analysis referred to all glycine substitutions associated with a lethal form of OI reported in the collagen type I genes. Their characteristics and distribution along *COL1A1* and *COL1A2* was performed to better understand the impact of lethal regions on clinical severity of mutations.

All calculations were carried out according to the protein position of the variants. If the mutation was reported to cause more than one type of OI, each clinical outcome of a particular mutation was counted as a separate variant. As the mortality of the lethal regions is predominantly linked with missense mutations, only glycine substitutions with a confirmed pathogenic status and a described OI type (I–IV) were analyzed.

## Results

In the tested cohort seven individuals presenting non-lethal OI phenotypes despite harboring a glycine substitution within lethal region of collagen type I genes were identified. Among six distinct variants, four are reported for the first time. The identification of non-lethal cases caused by mutations located in regions expected to result in the most severe form of OI, prompted us to investigate the dedicated OIVD. Data analysis of the deposited records was performed to determine the actual mortality of glycine substitutions mapping to these special gene regions and to investigate distribution and characteristics of all amino acids substituting for glycine in the entire *COL1A1* and *COL1A2* genes. Detailed information are provided in the corresponding sections, summarized in tables and presented in figures.

### Clinical Presentation of the Positive Cases

In the study population of 166 Polish patients suspected with OI, seven individuals were found to carry pathogenic glycine substitutions located within lethal regions of the collagen type I genes. The age of the patients, ranging between 3 and 25 years old, excluded the expected lethal outcome associated with localization of the causative variant. The individuals were diagnosed with mild OI type I, moderately deforming type IV or progressively deforming type III, and presented a broad spectrum of clinical features. The patients’ phenotype is summarized in Table 1.

**TABLE 1:** Clinical characteristics of patients reported in the study.

**Patients number**	**Age**	**Height (centile)**	**Prenatal fractures**	**Number of fractures**	**BS**	**DI**	**Hearing impairment**	**Long bone deformation**	**Face dysmorphia**	**Inheritance**	**Mobility**	**Bisphosphonate treatment**	**OI type**
1.	6	<3	Yes	Unknown	Yes	Yes	Suspected	Yes	Yes	Suspected parental mosaicism	Delayed	Yes	III
2.	7	<3	Yes	22	No	Yes	No	Yes	Yes	*De novo*	Wheelchair	Yes	III
3.	25	<3	Yes	20	Yes	Yes	No	Yes	Yes	*De novo*	Wheelchair	Yes	III
4.	23	<3	No	12	No	No	No	Yes	No	Suspected *de novo*	Clubfeet	Yes	IV
5.	3	<3	Yes	12	Yes	No	No	Yes	Yes	*De novo*	Impeded	Yes	III
6.	9	<3	Yes	10	No	No	No	Yes	Yes	Suspected *De novo*	Wheelchair	Yes	III
7.	18	25–50	No	7	Yes	No	No	No	No	*De novo*	Proper	No	I

*Abbreviation BS refers to blue sclera, DI to Dentinogenesis imperfecta.*

*Patient 1* is a 6-year-old girl, a second child of non-consanguineous parents, with features of OI noticeable after week 20 of pregnancy. After birth, the patient presented severe craniofacial dysmorphism and blue sclera. Shortened extremities, reduced muscle tone, and signs of numerous *in utero* fractures were observed. Further examination showed chest distortion, flattened vertebrae, delayed psycho-motor development, DI, and hearing impairment. After treatment with bisphosphonates initiated at week 3 of life, no further fractures were reported. A new c.2984G>C (p.Gly995Ala) mutation was identified in *COL1A1* gene in the girl. No such variant was observed in either parent by NGS or by targeted Sanger sequencing. However, targeted Sanger sequencing confirmed its presence in a biobanked blood sample from a deceased older brother. The first child, with perinatal fractures, circulatory difficulties, and severe phenotype, died at the age of 6 months. Due to observed recurrence, we suspect germinal mosaicism in one of the parents.

*Patient 2* is a 7-year-old girl, initially diagnosed with thanatophoric dysplasia with multiple bone defects observed in USG performed at week 21 of pregnancy. The ultrasound image revealed signs of perinatal fractures, poor skeleton mineralization, irregular width of ribs, shortened, and bent extremities, which changed the diagnosis to OI type IIB. Multiple facial deformities and features of DI were observed. The patient sustained about 20 fractures following birth. At the age of 3½ years, she was not able to walk independently: she is currently using a wheelchair. Treatment with bisphosphonates started at week 7 of life. Since then, two fractures have been reported. NGS sequencing revealed a novel c.2993G>C (p.Gly998Ala) mutation in the *COL1A1* gene. Family screening performed in parents excluded the presence of the variant.

*Patient 3* is a 25-year-old female diagnosed with OI type III with weight and body height deficiency, both below third centile. She has sustained about 20 fractures and is currently using a wheelchair. She presents features characteristic of the progressively deforming type, including shortening of lower extremities, barrel-shaped chest, disproportionately large head, triangular-shaped face, blue sclera, and DI. Pamidronate treatment started at the age of 15 years. The patient was found to harbor a novel c.1667G>T (p.Gly556Val) mutation in the *COL1A2* gene. The patient has a negative family history and healthy siblings. The mutation was not found in the mother by Sanger sequencing; however, as no DNA sample was available from the father, we can only suspect the *de novo* appearance of the mutation.

*Patient 4* is a 23-year-old man with 12 reported fractures, with the first affecting the clavicle, observed at day 6 of life. Later fractures involved the femur, phalanx, and vertebrae. OI diagnosis was confirmed after a histopathologic test at the age of 4 years. Despite receiving several surgical interventions, the patient suffers from biconcave vertebral deformations due to height reduction in the lumbar spine, and demonstrates an abnormal posture, round back, and asymmetry of the scapulae. The patient suffers from lower limb asymmetry, valgus knee and flat lopsided feet, and demonstrates a disturbed gait. Pamidronate treatment performed at the age of 12–16 equalized mineral bone density level and possibly reduced the number of fractures. A previously reported c.2539G>A (p.Gly847Ser) mutation was found to be present in the *COL1A2* gene. Segregation analysis was not performed in patient’s parents.

*Patient 5* is a 3-year-old girl with severe bone deformations detected by prenatal ultrasound at week 21 of gestation, suggesting lethal OI type II. The girl was found to have 11 intrauterine fractures of the extremities and ribs. Only one postnatal fracture was reported as pamidronate treatment was included in the neonatal period. After birth, a triangular-shaped face, soft skull bones, frontal bossing, blue sclera, upper and lower bone shortening, and deformities were observed. The patient harbors a known mutation c.2845G>A (p.Gly949Ser) in the *COL1A2* gene. Family diagnostics excluded the presence of the mutation in parents.

*Patient 6* is a 9-year-old girl. Pamidronate treatment was introduced during week 3 after birth. She has experienced 10 postnatal fractures of upper and lower limbs, as well as prenatal fractures of the femurs. The patient uses a wheelchair. She presents with a barrel-shaped chest and spinal defects, as well as shortening and bowing of limbs. Features characteristic for OI, such as skull deformations like triangular shape and frontal bossing, were observed. A c.2845G>A (p.Gly949Ser) mutation was found in the *COL1A2* gene, as in *Patient 5*, although the two probands are unrelated. Family history for OI is negative, parents were not tested as they were unavailable for family screening.

*Patient 7* is an 18-year-old man, presenting skeletal deformities such as a barrel-shaped chest, asymmetry of shoulders and scapulae, kyphosis, and disproportionate stature. Other extra-skeletal features like blue sclera, astigmatism, and joint hypermobility are also observed. The patient has suffered seven fractures of the upper and lower limbs and one vertebral fracture. Bone mineral density and *Z*-score values are below reference levels. Treatment with bisphosphonates was proposed. A novel c.3215G>T (p.Gly1072Val) mutation was identified in the *COL1A2* gene. The mutation was not observed in the parents, confirming the *de novo* appearance of the variant.

### Characteristics and Classification of the Identified Variants

A total of six distinct glycine substitutions located in the lethal regions of collagen type I genes were identified. Two variants located in lethal cluster 1 of the *COL1A1* gene resulted in glycine-to-alanine substitutions (p.Gly995Ala, p.Gly998Ala). In the *COL1A2* gene, pathogenic variants were identified in four out of eight lethal domains, resulting in glycine-to-serine (p.Gly847Ser, p.Gly949Ser) and glycine-to-valine (p.Gly556Val, p.Gly1072Val) substitutions.

According to the OIVD, two variants have been previously reported. The first variant p.Gly847Ser, located in lethal region 6, has been identified in three independent studies in patients classified with mild type I and moderate type IV. The second substitution p.Gly949Ser, located in lethal region 7, has been reported in nine patients, with lethal type II, borderline type II/III, and progressively deforming type III.

**FIGURE 2:**
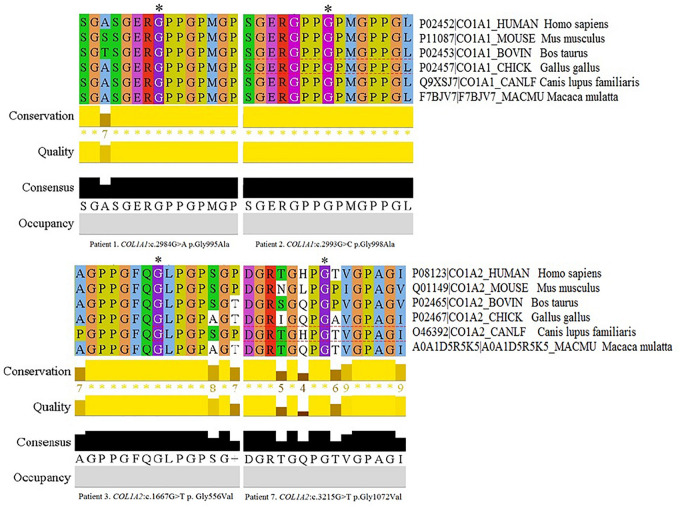
Multiple Sequence Alignment analysis of novel variants across several species in Jalview 2.11.0, Clustal Omega 1.2.4. The conservation calculation is based on AMAS method of multiple sequence alignment analysis with identities scoring highest, and amino acids with substitutions in the same physico-chemical class have the next highest score. The score for each column is shown below the histogram. The conserved columns with a score of 11 are indicated by “*”. Columns with a score of 10 have mutations but all conserved properties are marked with a “+”.

**FIGURE 3:**
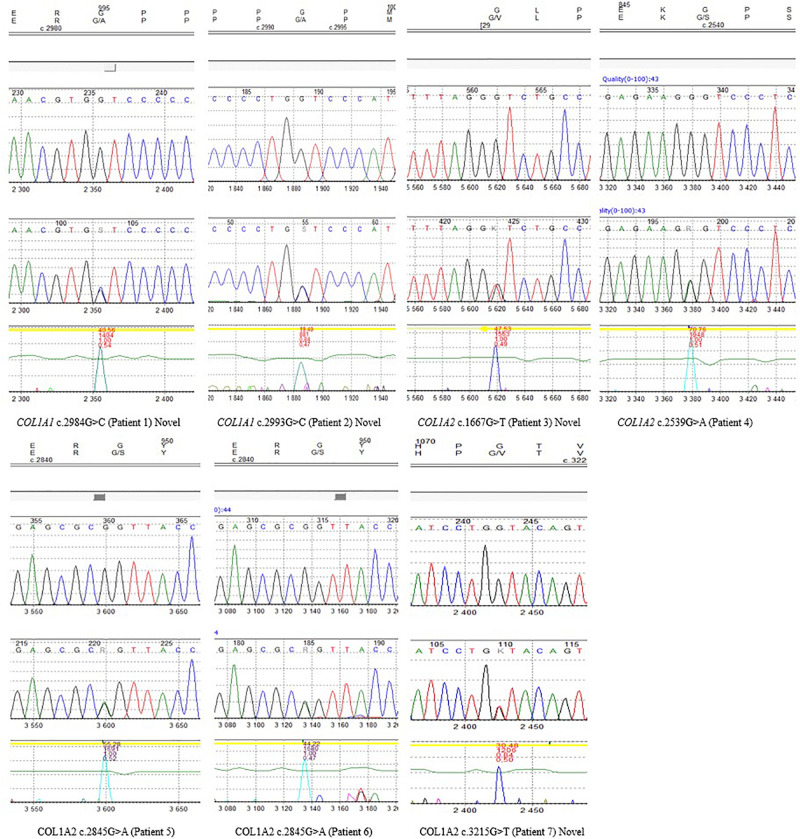
Sanger sequencing chromatograms of all identified variants reported in seven patients, including novel mutations.

The remaining four variants are reported for the first time. Two glycine-to-alanine mutations (p.Gly995Ala and p.Gly998Ala) were identified in exon 41, corresponding to the first lethal region of the *COL1A1* gene. In the *COL1A2* gene, two glycine-to-valine substitutions were reported in exon 29 (p.Gly556Val) and exon 48 (p.Gly1072Val); these correspond to the second and eighth lethal region, respectively. None of these variants has been reported in any human control or mutation database, including dbSNP, HGDM, ClinVar, or dedicated OIVD. No mutations have been found in the gnomAD Exomes (reaching good gnomAD Exome coverage, ranging from 69.9 to 82.5) or in gnomAD Genomes (reaching good gnomAD Genome coverage, ranging from 30.1 to 31.2). For all four variants, a deleterious effect on the gene or gene product was indicated by multiple lines of computational evidence of at least 10 pathogenic predictions (Varsome interpretation tool). According to the Multiple Sequence Alignment analysis all novel variants reached the highest conservation score across several species (Jalview 2.11.0, Clustal Omega 1.2.4; Figure 2). Finally, three variants were classified as likely pathogenic and one as pathogenic according to the ACMG classification. All novel mutations were submitted to the ClinVar database and assigned with accession numbers described in Table 2. Sanger sequencing chromatograms of the six identified mutations are presented in Figure 3.

**TABLE 2:** Variants reported in the study.

**Nucleotide position**	**Protein position (helical position)**	**Effect**	**Localization**	**Lethal region**	**Novel mutation**	**Accession number**	**Patients number**	**OI type**
***COL1A1***								
c.2984G>C	p.Gly995Ala (p.Gly817Ala)	Missense	exon 41	1	Yes	SCV000994662.1 (rs1598288656)	1	III
c.2993G>C	p.Gly998Ala (p.Gly820Ala)	Missense	exon 41	1	Yes	SCV000994663.1 (rs1598288634)	2	III
***COL1A2***								
c.1667G>T	p.Gly556Val (p.Gly466Val)	Missense	exon 29	2	Yes	SCV000994680.1 (rs1584322737)	3	III
c.2539G>A	p.Gly847Ser (p.Gly757Ser)	Missense	exon 40	6	Reported 3 times*	—	4	IV
c.2845G>A	p.Gly949Ser (p.Gly859Ser)	Missense	exon 44	7	Reported 9 times*	—	5 and 6	III
c.3215G>T	p.Gly1072Val (p.Gly982Val)	Missense	exon 48	8	Yes	SCV000994676.1 (rs1584330959)	7	I

*All variants were reported in a heterozygous state. ^∗^reported in Osteogenesis Imperfecta Variant Database (https://oi.gene.le.ac.uk/).*

### Review of all Glycine Substitutions Located Within Lethal Regions of *COL1A1* and *COL1A2* Genes

The review of all glycine substitutions mapping to the lethal clusters was prepared according to the records deposited in the dedicated OIVD. A total of 33 unique glycine substitutions in 41 patients have been reported in the two lethal regions corresponding to the Major Ligand-Binding Regions in the *COL1A1* gene. However, two variants resulted in at least two different OI types, expanding the number of mutations to 36 distinct clinical outcomes. In addition, 109 distinct glycine substitutions in 162 patients have been reported in the eight lethal regions identified in the *COL1A2* gene. Of all mutations, 15 resulted in at least two different OI types, increasing the number of phenotype-dependent variants to 126.

**FIGURE 4:**
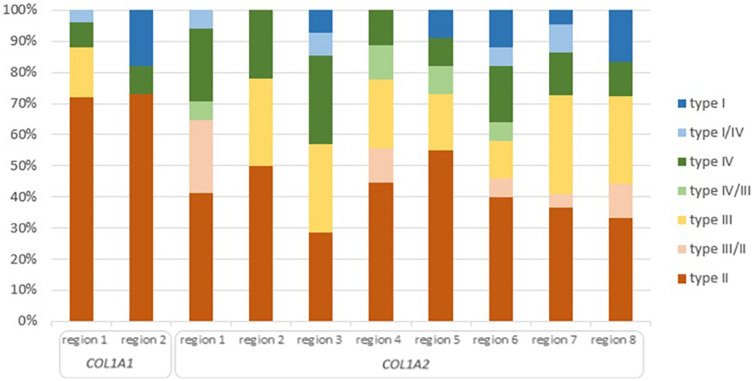
Clinical severity of glycine substitutions reported in the lethal regions of collagen type I genes. Each type of OI refers to different color seen in the legend. High of the bars reflect percentage participation of particular OI type in each lethal region.

Of the 36 distinct clinical outcomes of glycine substitutions reported in two lethal regions of *COL1A1* gene, 26 mutations (71%) resulted in a fatal outcome. Remaining mutations accounted for a non-lethal OI type III (*n* = 4), type IV (*n* = 3), type I (*n* = 2), and borderline type I/IV (*n* = 1; Figure 4). Although both regions presented a slightly different distribution of OI types, 70% of variants located in lethal region 1 (18 out of 25) and 72% in region 2 (8 out of 11) resulted in fatal form of the disease.

Seven amino acids have been found to substitute for glycine in lethal regions of the *COL1A1* gene. The occurrence and clinical severity of glycine residues varied between the two regions, with a total number of distinct mutations presented in the order: Ser (*n* = 9) > Ala (*n* = 8) > Asp, Cys (*n* = 7) > Glu, Val (*n* = 2) > Arg (*n* = 1). Each amino acid was responsible for at least one lethal case. The highest numbers of distinct OI type II mutations was reported in the following order: Ser (*n* = 8) > Cys (*n* = 7) > Asp (*n* = 5) > Ala, Val (*n* = 2) > Arg, Glu (*n* = 1; Table 3). Amino acids substituting for glycine have been associated with different ratio of lethal and non-lethal mutations. For *COL1A1* gene, all substitutions by arginine, cysteine, and valine resulted in fatal outcome in both regions. Substitutions by serine and aspartic acid caused OI type II phenotype in over two-thirds of variants; glutamic acid in half of cases, and alanine residues in around a quarter.

**TABLE 3:** Summary of the effects of substitution for glycine in lethal regions of the *COL1A1* and *COL1A2* gene with the number of mutations resulting in each type of Osteogenesis imperfecta.

**OI type/Amino acid**	**Ala**	**Arg**	**Asp**	**Cys**		**Glu**	**Ser**	**Val**	**Total**	**% participation of OI type**
***COL1A1* gene**										
I	1	–	–	–		1	–	–	2	6%
I/IV	1	–	–	–		–	–	–	1	3%
IV	3	–	–	–		–	–	–	3	8%
III/IV	–	–	–	–		–	–	–	0	0%
III	1	–	2	–		–	1	–	4	11%
II/III	–	–	–	–		–	–	–	0	0%
II	2	1	5	7		1	8	2	26	72%
Total	8	1	7	7		2	9	2	36	100%
Ratio of lethal vs non-lethal mutations	2:6	1:0	5:2	7:0		1:1	8:1	2:0	26:10	–

**OI type/Amino acid**	**Ala**	**Arg**	**Asp**	**Cys**	**Glu**	**Ser**	**Val**	**Phe**	**Total**	**% participation of OI type**

***COL1A2* gene**										
I	1	–	2	1	–	2	2	–	8	6,5%
I/IV	–	1	2	–	–	1	1	–	5	4%
IV	2	2	3	3	2	8	2	–	22	17,5%
III/IV	–	1	1	–	–	2	–	–	4	3%
III	1	3	4	2	–	12	5	–	27	21,5%
II/III	–	–	2	–	1	4	2	–	9	7%
II	–	5	18	6	3	9	9	1	51	40,5%
Total	4	12	32	12	6	38	21	1	126	100%
Ratio of lethal vs non-lethal mutations	0:4	5:7	18:14	6:6	3:3	9:29	9:12	1:0	51:75	–

*The odds the mutations being lethal for each residue is given.*

Of the 126 phenotype-dependent glycine substitutions reported in eight lethal regions of *COL1A2* gene, 51 mutations (40%) resulted in fatal outcome. Non-lethal variants have been associated with OI type III (*n* = 27), type IV (*n* = 22), type II/III (*n* = 9), type I (*n* = 8), type I/IV (*n* = 5), and III/IV (*n* = 4; Figure 4). Similarly to the lethal domains of *COL1A1*, frequency of particular OI types varied between the eight regions. The number of lethal variants was ranging between four distinct mutations in region 3, to nine mutations in region 2. Majority of variants located in lethal domains resulted in fatal outcome, with the exception of cluster 3, in which equal numbers type II, III, and IV OI have been recognized. The highest proportion of 55% mutations causing OI type II was observed in region 5 (6 out of 11), while the lowest of 28% in region 3 (4 out of 14).

Eight amino acids substituting for glycine in lethal clusters of *COL1A2* gene presented differences in frequency, with a total number of distinct mutations descending in the order: Ser (*n* = 38) > Asp (*n* = 32) > Val (*n* = 21) > Arg, Cys (*n* = 12) > Glu (*n* = 6) > Ala (*n* = 4) > Phe (*n* = 1). Data analysis revealed variable clinical severity of glycine residues. Number of distinct variants leading to OI type II descends in the following order: Asp (*n* = 18) > Ser, Val (*n* = 9) > Cys (*n* = 6) > Arg (*n* = 5) > Glu (*n* = 3) > Phe (*n* = 1; Table 3). For *COL1A2* gene, phenylalanine reached the highest mortality, however, the substitution has been reported in a single case of lethal OI only. Aspartic acid, cysteine, glutamic acid, valine, and arginine substitutions resulted in nearly equal numbers of lethal and non-lethal phenotypes, while only one-quarter of serine substitutions resulted in a fatal outcome. All reported alanine substitutions have been non-lethal and resulted in OI type I (*n* = 1), IV (*n* = 2), and III (*n* = 1).

### Review of all Glycine Substitutions Leading to Lethal Outcome and Their Distribution Along *COL1A1* and *COL1A2* Genes

The review of OIVD records showed that 34% of glycine substitutions (154 mutations) in *COL1A1* and 17% (80 mutations) in *COL1A2* resulted in OI type II. So far, lethal variants have been reported in 38 and 30 of out 52 exons comprising *COL1A1* and *COL1A2* gene, respectively. Their distribution along alpha(I) chains is not regular, however, a marked increase of lethal mutations was observed from exon 17 toward the 3’end in both genes.

Lethal regions have been described according to the amino acid position. Although they are located within eight exons of *COL1A1* and nineteen of *COL1A2*, they do not always cover the entire exon. Compared to the length of helical domains of collagen type I genes, which are both 1,014 amino acids long, the two lethal clusters of the *COL1A1* gene comprise 186 amino acids (18%), while the eight clusters of *COL1A2* comprise 315 amino acids (31%).

**FIGURE 5:**
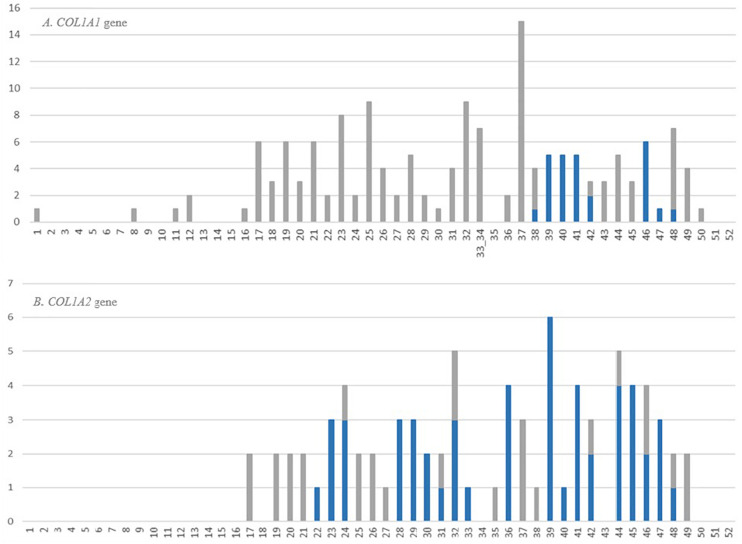
Number and distribution of distinct lethal glycine substitutions along the alpha1 chain of the **(A)** *COL1A1* and **(B)** *COL1A2* genes. The vertical axis shows number of reported mutations, and the horizontal axis shows the gene exons. The bars refer to the number of distinct lethal variants reported in a particular exon. The gray bars represent mutations located outside the lethal regions, blue bars represent mutations corresponding to the location of lethal regions.

In the *COL1A1* gene, the highest number of distinct glycine substitutions leading to a fatal outcome, was reported in exon 37 (*n* = 15), located right before lethal region 1 (MLBR2). Among the exons corresponding to lethal clusters, a maximum of six variants causing OI type II was found in exon 46 mapping entirely to lethal region 2 (MLBR3). In the *COL1A2* gene, the exons within the lethal domains demonstrated elevated numbers of lethal mutations. The highest number of distinct variants leading to a fatal outcome (*n* = 6) was reported in exon 39, belonging to the lethal cluster 5. Exons located outside the lethal regions have been associated with maximum of only three distinct lethal variants (Figure 5).

The frequency and severity of amino acids substituting for glycine shows variable pattern depending on gene in which mutation occurs. In the *COL1A1*, seven glycine residues have been reported to cause OI type II, with a number of distinct variants descending in the following order: Asp (*n* = 34) > Ser (*n* = 31) > Arg (*n* = 29) > Val (*n* = 25) > Cys (*n* = 23) > Glu (*n* = 7) > Ala (*n* = 5). Regarding proportion of lethal and non-lethal mutations valine, which is the only hydrophobic amino acid substituting for glycine in the *COL1A1* gene, presented the highest lethality, reaching 64% (25 out of 39 mutations). Residues with charged side chains (Asp, Glu, Arg) resulted in approximately equal numbers of lethal and non-lethal variants, while small amino acids (Cys, Ser, Ala) resulted in fatal outcome in one-third of the mutations (Table 4).

**TABLE 4:** Summary of amino acids substituting for glycine in *COL1A1* and *COL1A2* gene resulting in a lethal outcome.

	***COL1A1* gene**			***COL1A2* gene**		
**Amino acid**	**Number of non-lethal mutations**	**Number of lethal mutations**	**Total number of mutations**	**Number of non-lethal mutations**	**Number of lethal mutations**	**Total number of mutations**
Ala	38	5	43	13	0	13
Arg	38	29	67	58	8	66
Asp	28	34	62	77	25	102
Cys	50	23	73	39	7	46
Glu	9	7	16	26	9	35
Leu	0	0	0	0	1	1
Phe	0	0	0	0	1	1
Ser	121	31	152	111	10	121
Val	14	25	39	60	19	79
Total	298	154	452	384	80	464

*The ratio of lethal mutations is given.*

In the *COL1A2* gene, eight amino acids have been associated with OI type II. The number of distinct mutations resulting in a lethal phenotype is presented in the following order: Asp (*n* = 25) > Val (*n* = 19) > Ser (*n* = 10) > Glu (*n* = 9) > Arg (*n* = 8) > Cys (*n* = 7) > Leu, Phe (*n* = 1) > Ala (*n* = 0). According to the odds of lethal variants compared to the number of all mutations reported in the *COL1A2* gene, hydrophobic amino acids (Leu, Phe) have been associated with fatal outcomes only; however, single variants have been reported. Mutations resulting from acidic charged (Glu, Asp) and hydrophobic (Val) residues have been lethal in one-quarter of substitutions. Small amino acids (Cys, Ser) and positively charged arginine resulted in a fatal manifestation in 15% of cases, while substitutions by alanine were only associated with non-lethal phenotypes (Table 4).

## Discussion

Lethal regions are special domains located in the collagen type I genes associated with the presence of glycine substitutions leading to the most severe, lethal form of OI. These special gene regions correspond to the localization of functional domains responsible for intramolecular interactions of collagen molecules. In the population-based study of 166 individuals with OI, we identified six pathogenic variants located in the lethal regions of the *COL1A1* and *COL1A2* genes in seven patients presenting a non-lethal phenotypes. Of these mutations, four are reported for the first time.

Genotype–phenotype analysis, based on data available in the OIVD, revealed that clinical outcome of the identified novel variants corresponds to severity observed in similar cases. In lethal region 1 of the *COL1A1* gene, two novel alanine substitutions (p.Gly995Ala and p.Gly998Ala) were identified in two patients classified with progressively deforming type III. From 23 distinct glycine substitutions reported in this region, only two were replaced by alanine: one resulted in moderate type IV, and the other with borderline type I/IV, moderate type IV, and progressively deforming type III (Osteogenesis Imperfecta Variant Database, 2020). In the *COL1A2* gene, three patients were found to harbor a causative glycine-to-serine substitution (p.Gly847Ser and p.Gly949Ser). The first variant (p.Gly847Ser), located in lethal region 6, was identified in a single patient presenting a moderate phenotype. The mutation has previously been reported in three independent studies where patients were classified with OI type I and IV. Serine is the most common amino acid substituting for glycine in this region; most variants resulted in a moderate and mild outcomes, and only one lethal mutation has been reported (Osteogenesis Imperfecta Variant Database, 2020). The second serine substitution (p.Gly949Ser), located in lethal region 7, was identified in two unrelated patients, both diagnosed with progressively deforming type III. The variant has previously been reported in nine patients, with lethal type II, borderline type II/III, and progressively deforming type III (Osteogenesis Imperfecta Variant Database, 2020). In the study group, two individuals were found to harbor novel glycine to valine substitutions (p.Gly556Val and p.Gly1072Val); these were associated with the progressively deforming and mild forms of OI, respectively. The first variant (p.Gly556Val) was identified in lethal region 2, where only three valine substitutions have previously been reported: one was identified in a single patient with OI type III, while the other two resulted in OI type II in three patients (Osteogenesis Imperfecta Variant Database, 2020). The second mutation (p.Gly1072Val) was localized in lethal region 8. Similarly to region 2, only three valine substitutions have been reported; these resulted in single cases of mild type I, borderline type II/III, and lethal type II (Osteogenesis Imperfecta Variant Database, 2020).

The results of this primary analysis prompted us to perform a systematic review of all variants reported in the lethal regions to determine their actual mortality. At the time when these special gene domains were distinguished, glycine substitutions located in lethal clusters of the alpha1(I) chain were almost exclusively fatal. In turn, 86% of mutations mapping to lethal regions of the alpha2(I) chain resulted in type II (Marini et al., 2012). Based on the current dataset of OI mutations, the mortality associated with variants located in the lethal regions reaches 72% for *COL1A1* and 40% for *COL1A2* (Osteogenesis Imperfecta Variant Database, 2020).

A short analysis of all glycine substitutions leading to OI type II and their distribution along collagen type I genes, revealed that lethal variants have been reported in more than a half of the exons comprising both collagen type I genes (Osteogenesis Imperfecta Variant Database, 2020). Interestingly a total of 26 (17%) fatal glycine substitutions located in *COL1A1* and 51 (64%) in *COL1A2*, corresponds to location of the lethal regions. Thus for *COL1A1* gene, a significant number of variants leading to a fatal outcome appears to be located outside lethal domains. It is possible that the structural aberrations situated before the ligand-binding regions may indirectly affect interactions between collagen and extracellular molecules. The small number of lethal variants found to be linked with lethal regions in the *COL1A1* gene may be also due to the random occurrence of OI mutations and the fact that only 18% of the helical domain of the alpha1(I) chain is covered by lethal regions. Alternatively, there might be other factors influencing the high mortality in other parts of the *COL1A1* gene. On the contrary, in the *COL1A2* gene, a fatal outcome is more strongly linked with localization in regions of intermolecular interactions. Although the eight lethal regions constitute only 31% of the alpha2(I) chain, it appears that the proteoglycan binding-sites have a marked impact on overall mortality.

Despite the fact that lethality values are lower than previously calculated, glycine substitutions generally are associated with higher mortality when located in lethal clusters, except for alanine in the *COL1A2* gene. According to previous studies, substitutions by hydrophobic valine and charged amino acids are more likely to lead to a fatal outcome than substitutions by small amino acids (Marini et al., 2012). Interestingly, substitutions by alanine, cysteine and serine result in lethal phenotype more often when located in lethal regions. This observation is consistent with the model proposed by Bodian et al. (2008), especially for lethal clusters of the *COL1A1* gene. Therefore, it appears that ligand-binding sites may influence the clinical severity of mutations; however, the extent varies not only between amino acids but also depends on gene and particular lethal region in which the substitution occurs. Perhaps, observed differences may be a result of the distinct roles of each chain and region in intermolecular interactions, as well as variable effect of the substituting residue on collagen structure. It is possible that some of the resulting structural aberrations of the ligand binding-sites are more or less tolerated by particular extracellular molecules, resulting in a spectrum of lethal and non-lethal outcomes.

According to the primary assumptions, the localization of a mutation in a lethal region was believed to lead to a fatal manifestation, regardless of which amino acid was substituting for glycine (Marini et al., 2012). However, our findings indicate that the amino acids that replace the glycine in fact have a considerable influence on the clinical outcome of the mutation. Additionally, recurrence of mutation at a single glycine position have also been reported in lethal clusters of the *COL1A2* gene. The vast majority of cases resulted in a range of OI types, with the severity depending on the substituting amino acid. Moreover, substitutions by the same amino acid in different patients resulted in differing non-lethal phenotypes, as well as lead to intermixed lethal and non-lethal cases, as observed in *Patient 1* and her brother (Osteogenesis Imperfecta Variant Database, 2020). This suggest that final clinical outcome of mutations located in lethal regions could be modified not only by amino acid *per se* but also by additional factors (Bodian et al., 2008; Xiao et al., 2015; Qiu et al., 2018).

On the basis of the current dataset of OI mutations, evaluation of the lethal regions’ impact on clinical severity remains an elusive task. Although a limited number of available variants does not allow for a reliable genotype–phenotype correlation studies, presented paper provides a relevant information for a healthcare professionals. Perhaps increasing number of reported variants will in future allow more precise identification of the truly exclusively lethal sites within collagen type I genes. Till then, we believe that in the case of patients with confirmed pathogenic mutations located in lethal regions, the term “lethal” should not be used in genetic counseling or when interpreting molecular test results. We agree with other authors that mutations mapping to a lethal domains should not be the deciding factor, especially in prenatal counseling (Rauch et al., 2010; Kang et al., 2017). Clinicians should be aware that final outcome of such variants may result in a non-lethal, albeit severe, phenotype, or even a mild clinical manifestation. A molecular report alone indicating a pathogenic variant in the ligand binding region is not sufficient to predict the severity of the phenotype and clinical manifestation. However, this finding may suggest a potentially fatal outcome when correlated with a severe perinatal ultrasound image. Thus, timely genetic analysis may help to define the most appropriate perinatal care or treatment.

## Data Availability Statement

The sequencing data of four novel variants that we describe in the manuscript were deposited in ClinVar database that is directly linked with dbSNP database. For each variant a separate number is provided and listed in Table 2 as “rs number” and “accession number”. The two variants that have been previously-reported were already added by other authors and are available in public databases (e.g., dbSNP, ClinVar, OIVD). The datasets generated for this study can be found in the Osteogenesis Imperfecta Variant Database v2.0 website (https://oi.gene.le.ac.uk/home.php). Although the OIVD v2.0 has been frozen, it has been recently converted to the Global Variome shared LOVD v3.0. The previous version that has been used to prepare data analysis sections is still available for users.

## Ethics Statement

The studies involving human participants were reviewed and approved by Polish Mother’s Memorial Hospital Research Institute Ethics Committee. Written informed consent to participate in this study was provided by the participants’ legal guardian/next of kin. Written informed consent was obtained from the individual(s) for the publication of any potentially identifiable images or data included in this article.

## Author Contributions

AG and KS: conceptualization. KS: formal analysis, writing-original draft preparation, visualization, and project administration. KS, IP, LR, and DS: investigation. AG, DC-S, EJ-P, IM, ŁK, AJ, EB-O, and IJ: resources. AG: writing-review and editing. AG and LJ: supervision. KS and AG: funding acquisition. All authors agreed to be accountable for the content of the work.

## Conflict of Interest

The authors declare that the research was conducted in the absence of any commercial or financial relationships that could be construed as a potential conflict of interest.

## Footnotes

1 https://varsome.com/

2 https://oi.gene.le.ac.uk/home.php

## Abbreviations

OI: Osteogenesis imperfecta
DI: Dentinogenesis imperfecta
MLBR: Major Ligand-Binding Regions
NGS: Next Generation Sequencing
OIVD: Osteogenesis imperfecta Variant Database.

## References

[B1] Basel D. Steiner R. D. (2009). Osteogenesis imperfecta: recent findings shed new light on this once well-understood condition. *Genet. Med.* 11 375–385. 10.1097/GIM.0b013e3181a1ff7b 19533842

[B2] Bodian D. L. Chan T. F. Poon A. Schwarze U. Yang K. Byers P. H. (2009). Mutation and polymorphism spectrum in osteogenesis imperfecta type II: implications for genotype-phenotype relationships. *Hum. Mol. Genet.* 18 463–471. 10.1093/hmg/ddn374 18996919 PMC2638801

[B3] Bodian D. L. Madhan M. Brodsky B. Klein T. E. (2008). Predicting the clinical lethality of osteogenesis imperfecta from collagen glycine mutations. *Biochemistry* 47 5424–5432. 10.1021/bi800026k 18412368

[B4] Byers P. H. Wallis G. A. Willing M. C. (1991). Review article Osteogenesis imperfecta: translation of mutation to phenotype. *J. Med. Genet.* 28 433–442. 1895312 10.1136/jmg.28.7.433 PMC1016951

[B5] Cole W. G. Dalgleish R. (1995). Perinatal lethal osteogenesis imperfecta. *J. Med. Genet.* 32 284–289. 7643358 10.1136/jmg.32.4.284 PMC1050377

[B6] Di Lullo G. A. Sweeney S. M. Körkkö J. Ala-Kokko L. San Antonio J. D. (2002). Mapping the ligand-binding sites and disease-associated mutations on the most abundant protein in the human, type I collagen. *J. Biol. Chem.* 277 4223–4231. 10.1074/jbc.M110709200 11704682

[B7] Forlino A. Cabral W. A. Barnes A. M. Marini J. C. (2012). New perspectives on osteogenesis imperfecta. *Nat. Rev. Endocrinol.* 7 540–557. 10.1038/nrendo.2011.81 21670757 PMC3443407

[B8] Forlino A. Marini J. C. (2016). Osteogenesis imperfecta. *Lancet* 387 1657–1671. 10.1016/S0140-6736(15)00728-X 26542481 PMC7384887

[B9] Hruskova L. Fijalkowski I. Van Hul W. Marik I. Mortier G. Martasek P. (2016). Eight mutations including 5 novel ones in the COL1A1 gene in Czech patients with osteogenesis imperfecta. *Biomed. Pap.* 160 442–447. 10.5507/bp.2016.022 27132807

[B10] Kang H. Aryal S. Marini J. C. (2017). Osteogenesis imperfecta: new genes reveal novel mechanisms in bone dysplasia. *Transl. Res.* 181 27–48. 10.1016/j.trsl.2016.11.005 27914223

[B11] Lindahl K. Åström E. Rubin C. J. Grigelioniene G. Malmgren B. Ljunggren O. (2015). Genetic epidemiology, prevalence, and genotype–phenotype correlations in the Swedish population with osteogenesis imperfecta. *Eur. J. Hum. Genet.* 23 1042–1050. 10.1038/ejhg.2015.81 25944380 PMC4795106

[B12] Maasalu K. Nikopensius T. Kõks S. Nõukas M. Kals M. Prans E. (2015). Whole-exome sequencing identifies de novo mutation in the COL1A1 gene to underlie the severe osteogenesis imperfecta. *Hum. Genomics* 9:6. 10.1186/s40246-015-0028-0 25958000 PMC4429824

[B13] Maioli M. Gnoli M. Boarini M. Tremosini M. Zambrano A. Pedrini E. (2019). Genotype–phenotype correlation study in 364 osteogenesis imperfecta Italian patients. *Eur. J. Hum. Genet.* 27 1090–1100. 10.1038/s41431-019-0373-x 30886339 PMC6777444

[B14] Marini J. C. Blissett A. R. (2013). New genes in bone development: what’s new in osteogenesis imperfecta. *J. Clin. Endocrinol. Metab.* 98 3095–3103. 10.1210/jc.2013-1505 23771926 PMC3733862

[B15] Marini J. C. Forlino A. Bächinger H. P. Bishop N. J. Byers P. H. De Paepe A. (2017). Osteogenesis imperfecta. *Primer* 3:17052. 10.1038/nrdp.2017.52 28820180

[B16] Marini J. C. Forlino A. Cabral W. A. Barnes A. M. San Antonio J. D. Milgrom S. (2012). Consortium for osteogenesis imperfecta mutations in the helical domain of type i collagen: regions rich in lethal mutations align with collagen binding sites for integrins and proteoglycans. NIH Public Access. *Nat. Rev. Endocrinol.* 7:540. 10.1002/humu.20429 17078022 PMC4144349

[B17] Marini J. C. Lewis M. B. Wang Q. Chen K. J. Orrison B. M. (1993). Serine for glycine substitutions in type I collagen in two cases of type IV osteogenesis imperfecta (OI). Additional evidence for a regional model of OI pathophysiology. *J. Biol. Chem.* 268 2667–2673. 8094076

[B18] Mortier G. R. Cohn D. H. Cormier-Daire V. (2019). Nosology and classification of genetic skeletal disorders: 2019 revision. *Am. J. Med. Genet. Part A* 179A 2393–2419. 10.1002/ajmg.a.61366 31633310

[B19] Mouna Ben Amor I. Glorieux F. H. Rauch F. (2011). Genotype-phenotype correlations in autosomal dominant osteogenesis imperfecta. *J. Osteoporosis* 2011:540178. 10.4061/2011/540178 21912751 PMC3170785

[B20] Online Mendelian Inheritance in Men (OMIM) (2021). *Online Mendelian Inheritance in Men. Osteogenesis Imperfecta.* Available online at: https://www.omim.org (accessed date May, 2021).

[B21] Osteogenesis Imperfecta Variant Database. (2020). *Osteogenesis Imperfecta Variant Database (Part of the Leiden Open Variation Database). COL1A1.* Available online at: https://oi.gene.le.ac.uk/home.php?select_db=COL1A1 (accessed January, 2020). COL1A2. Avaialble online at: https://oi.gene.le.ac.uk/home.php?select_db=COL1A2 (accessed January, 2020).

[B22] Pollitt R. McMahon R. Nunn J. Bamford R. Afifi A. Bishop N. (2006). Mutation analysis of COL1A1 and COL1A2 in patients diagnosed with osteogenesis imperfecta type I-IV. *Hum. Mutat.* 27:716. 10.1002/humu.9430 16786509

[B23] Qiu Y. Mekkat A. Yu H. Yigit S. Hamaia S. Farndale R. W. (2018). Collagen Gly missense mutations: effect of residue identity on collagen structure and integrin binding. *J. Struct. Biol.* 203 255–262. 10.1016/j.jsb.2018.05.003 29758270 PMC6089640

[B24] Rauch F. Lalic L. Roughley P. Glorieux F. H. (2010). Genotype-phenotype correlations in nonlethal osteogenesis imperfecta caused by mutations in the helical domain of collagen type I. *Eur. J. Hum. Genet.* 18 642–647. 10.1038/ejhg.2009.242 20087402 PMC2987338

[B25] Reuter M. S. Schwabe G. C. Ehlers C. Marschall C. Reis A. Thiel C. (2013). Two novel distinct COL1A2 mutations highlight the complexity of genotype phenotype correlations in osteogenesis imperfecta and related connective tissue disorders. *Eur. J. Med. Genet.* 56 669–673. 10.1016/j.ejmg.2013.10.002 24140640

[B26] Robinson M. E. Rauch F. (2019). Mendelian bone fragility disorders. *Bone* 126 11–17. 31039433 10.1016/j.bone.2019.04.021

[B27] Steiner R. D. Basel D. (2019). “*COL1A1/2* osteogenesis imperfecta. 2005 (updated 2019 December 12),” in *GeneReviews^®^ [Internet]*, eds Adam M. P. Ardinger H. H. Pagon R. A. Wallace S. E. Bean L. J. H. Mirzaa G. (Seattle, WA: University of Washington, Seattle), 1993–2019. 20301472

[B28] Sweeney S. M. Orgel J. P. Fertala A. McAuliffe J. D. Turner K. R. Di Lullo G. A. (2008). Candidate cell and matrix interaction domains on the collagen fibril, the predominant protein of vertebrates. *J. Biol. Chem.* 283 21187–21197. 10.1074/jbc.M709319200 18487200 PMC2475701

[B29] Trejo P. Rauch F. (2016). Osteogenesis imperfecta in children and adolescents—new developments in diagnosis and treatment. *Osteoporos Int.* 27 3427–3437. 10.1007/s00198-016-3723-3 27492436

[B30] Van Dijk F. S. Sillence D. O. (2014). Osteogenesis imperfecta: clinical diagnosis, nomenclature and severity assessment. *Am. J. Med. Genet. Part A* 164A 1470–1481. 24715559 10.1002/ajmg.a.36545 PMC4314691

[B31] Wang Q. Orrison B. M. Marini C. (1993). Two additional cases of osteogenesis imperfecta with substitutions for glycine in the a2 (I) collagen chain. *J. Biol. Chem.* 268 25162–25167. 7693712

[B32] Xiao J. Yang Z. Sun X. Addabbo R. Baum J. (2015). Local amino acid sequence patterns dominate the heterogeneous phenotype for the collagen connective tissue disease Osteogenesis Imperfecta resulting from Gly mutations. *J. Struct. Biol.* 192 127–137. 10.1016/j.jsb.2015.05.002 25980613 PMC4898063

[B33] Zhytnik L. Maasalu K. Pashenko A. Khmyzov S. Reimann E. Prans E. (2019). COL1A1/2 Pathogenic variants and phenotype characteristics in ukrainian osteogenesis imperfecta patients. *Front. Genet.* 10:722. 10.3389/fgene.2019.00722 31447884 PMC6696896

[B34] Zhytnik L. Maasalu K. Reimann E. Prans E. Kõks S. Märtson A. (2017). Mutational analysis of COL1A1 and COL1A2 genes among Estonian osteogenesis imperfecta patients. *Hum. Genomics* 11:19. 10.1186/s40246-017-0115-5 28810924 PMC5558703

